# Toward stakeholders’ understanding of media reporting on doctor–patient relationship issues: trust, unfamiliarity and uncertainty in the Chinese context

**DOI:** 10.3389/fpubh.2024.1484828

**Published:** 2024-11-20

**Authors:** Zhenghan Gao, Junyang Huang, Bowen Zhang, Xinwen Zhang

**Affiliations:** ^1^School of Humanities and Foreign Languages, Qingdao University of Technology, Qingdao, China; ^2^School of Media, Film and Television, Jiangsu Normal University, Xuzhou, China; ^3^School of Business, Shenzhen Technology University, Shenzhen, China; ^4^Shenzhen Mental Health Centre, Shenzhen, China

**Keywords:** doctor–patient relationship, citizen journalism, hospital, audience, public health

## Abstract

**Introduction:**

This study explores doctors’ and patients’ understandings of citizen journalism on doctor–patient relationship issues. It also examines the communication effect of citizen journalism as a communication platform on doctors and patients who are taking part in the doctor–patient relationship in contemporary China.

**Method:**

This study draws on the analysis of 24 semi-structured interviews with doctors from both publicly funded and privately operated hospital, and nine focus groups which included 36 patients with different socio-economic backgrounds.

**Result:**

The empirical research present the following results: (1) authority and witness are the two key factors to construct the stakeholders’(doctors’ and patients’) awareness and trust of citizen journalist reporting on the doctor–patient relationship issues. (2) stakeholders’ perception on citizen journalism will construct them concern on the uncertain and unfamiliar knowledge during the hospital activities.

**Discussion:**

The interpretation of doctor–patient relationship reports by doctors and patients affects their mutual trust. Authority and witnessing are two key factors that citizen journalists should consider when reporting on doctor–patient relationship news. Doctors from different types of hospitals and patients with different income levels have different understandings of the authority and witnessing of the reported content. Reading the content of doctor–patient conflicts reflected in citizen journalist reports can exacerbate the emotional fluctuations of doctors and patients. After reading these reports, doctors and patients may experience increased anxiety about uncertainty and unfamiliarity in doctor–patient communication.

**Contribution:**

This study provides a framework for public health research from the relationship between communication content and audience. It also provides answers from the perspectives of media and stakeholders to investigate the tension in doctor–patient relationships in China.

## 1 Introduction

The doctor–patient relationship is one of the most significant public health subjects. Since the country’s founding, China’s healthcare reforms have profoundly impacted the doctor–patient relationship. Media attention has been drawn to the numerous doctor–patient confrontations in China in recent years. For example, in 2024, a cardiologist at the First Affiliated Hospital of Wenzhou Medical University in Wenzhou City, Zhejiang Province was killed by a patient; in 2020, an ophthalmologist at Beijing Chaoyang Hospital was stabbed by a patient; and in 2018, a fight broke out between doctors and patients at Beijing Jishuitan Hospital. These reports have also sparked heated debates among the general public. This essay examines how doctors and patients, as stakeholders, understand citizen journalism media coverage. It contends that core audiences with varying socioeconomic and cultural backgrounds interpret and comprehend these citizen journalist reports differently.

Since the beginning of the 21st century, the frequency of violent patient assaults on doctors in China has continued to rise. These disputes rose at an average yearly rate of 22.9% between 2002 and 2012 (1). In 2012, patients across the nation murdered 10 medical personnel. This number was fivefold more in 2011 and tenfold greater in 2010 (2). Since 2010, Chinese society has used the phrase *yinao* (Chinese: 医闹; pinyin:yī nào) to describe the destruction of the doctor–patient relationship by patients and their families. *Yinao* aims to describe the illegal attacks on doctors and hospitals taken by patients and their families because of their dissatisfaction with the doctor’s diagnosis and treatment (3). Patients and their families have been known to verbally trash the reputation of hospitals and physicians. It also includes patients and their families assaulting doctors illegally, threatening doctors’ lives, and trashing hospital property. The rationale behind yinao is the hope that victims and their families may receive greater medical compensation as a result of these attacks. According to the preceding description, yinao encompasses the violent and damaging behavior of patients toward doctors. However, this definition does not cover specific patient losses caused by medical and hospital malpractice. Based on Hesketh et al. (3) and Pan et al. (2), Yinao emphasizes the inflammatory protest behavior of patients and their families against doctors or hospital institutions due to dissatisfaction, but does not include the impact of doctors’ technical negligence on patients and the threat to the doctor–patient relationship. According to He (4) and Pan et al. (2), physicians are also to blame for the strained doctor–patient relationship in China. As a result of marketization medical reforms, the proliferation of overdiagnoses and overprescribing has led some physicians to engage in price gouging through medical diagnostic activities. This has harmed the profession of medicine. Some doctors may threaten the health of their patients as a result of treatment-related errors. It has altered society’s opinion of medicine as a profession. The condition under which society valued physicians has changed. This has caused some doctors to lose morale and become insensitive to the needs of their patients, leading to a rise in patient dissatisfaction. Zhou and Grady (5) coined the term doctor–patient conflict to describe these types of disagreements between doctors and patients. Doctor–patient conflict encompasses not only the yinao behavior of patients and their families toward doctors and hospitals, but also the problems between doctors and patients resulting from the carelessness of some doctors. According to Zhou and Grady (5), reporter and audience interest in doctor–patient conflict is the highest when the media reports on doctor–patient relationship concerns. Regarding the doctor–patient connection, a doctor–patient disagreement might be considered evidence of a broken doctor–patient relationship. In contrast, the frequent incidence of doctor–patient confrontations has intensified the ongoing tension between doctors and patients in modern China. This essay utilizes the results of the data analysis of qualitative semi-structured interviews with doctors and focus groups with patients to demonstrate how doctors and patients interpret citizen journalism reports on doctor–patient relationship concerns. It describes the essential criteria for establishing confidence in the reporting. Also discussed are the concerns of various groups on the unfamiliar and ambiguous information presented in the study.

## 2 Media reporting on public health issues

Everyone requires good health for survival and development. WHO defines health as “a state of complete physical, mental, and social wellbeing” (6), p. 558. According to Jackson (7), the media is the vehicle for disseminating information regarding public health, and the job of media reporting in public health is to integrate medical/health field knowledge with citizen understanding of health issues.

Mass media plays a mediating role in alleviating public health panic and enhancing the effectiveness of public health communication. Valente and Saba (8) assert that study through the lens of mass media and communication has been a crucial aspect of public health communication. This is due to the fact that the goal of the media is to promote public health, which cannot be divorced from the tendency that civic society should protect the rights and interests of individuals. In the process of promoting public health in the 21st century, Lupton (9) believes that the media’s propensity plays a crucial role. According to Seale (10), the media are essential for transmitting and spreading health information. Reporting on health issues in the mass media can improve the mental and physical health of the audience, minimize sickness panic, and increase social cohesiveness. Health-related information and imagery, such as lifestyle choices, products, and practices, may be disseminated through the mass media to the target audience. The mass media are not a sign that public health experts should be challenged, but they can be viewed as a communication tool upon which public health professionals rely to participate in promoting public health. According to Lefebvre (11), mass media function as communication bridges to facilitate the transmission of health information from health specialists to the general public.

Mass media can influence the audience’s health cognition and play a constructive role in shaping the public’s health literacy through the agenda setting of media content. Scholars that specialize in audience research have also backed the notion that mass media influences viewers’ perceptions of health. According to Wright (12), mass media reporting is the primary source of the audience’s interest in health information, particularly disease information related to personal life advantages and wellbeing. How individuals face these ailments in their life is determined by their unique interpretation of health messages from the mass media. Avery (13) performed 400 random phone interviews with American residents. Avery (13) argues that the reporting and dissemination of health information by the mass media influence both the health literacy of the audience and the enrichment of health information. Therefore, mass media should focus on comprehending the actual demands and viewing preferences of their audience for health information. Consequently, these reports can serve as an important means for the public to participate in personal health management.

According to Leask et al. (14), the notion of public health emphasizes the concern for the general health of various social groups. Public health policymakers must disseminate healthcare knowledge to the general population. Radio, television, and newspapers have devoted attention to public health issues in the period of mass media. These media outlets have contributed to the promotion of illness prevention, the extension of life, and the enhancement of the public’s sense of wellbeing (15). Agenda-setting by the mass media at the level of communication content can decide the aspects and actions they like the public to take regarding health issues. This type of communication involvement with audiences, promoting the public health information conveyed by mass media, becomes, to some extent, the public’s common impression (16).

However, the media’s entertaining and dramatic narratives on health topics create a gap between transmitted material and objective reality, which may cause the public to develop biases regarding health issues. According to Wright et al. (12), although the media have the ability to affect people’s health behaviors, they have negative consequences that contribute to the public having poor eating habits, drug usage issues, etc. In the era of mass media, audiences rely significantly on professional news media for the most up-to-date and accurate information regarding health issues; nevertheless, news media have their own agendas when providing health-related news. These agendas and covered topics may not reflect the actual situation. The agenda may only accommodate the development interests of the mass media or the specific desires of the interest groups it represents (17). In addition, mass media information collection is limited by time and space, which may result in inaccurate health news reporting (18).

Mass media may exacerbate the disparities between society and political power in regards to health. According to Lewis and Lewis (19), the most prevalent diseases in the media are not necessarily the most prevalent in reality. For instance, in magazine and news media, AIDS-related information is far more prevalent than cancer and cardiovascular disease coverage (20). Occasionally, the media may stigmatize the sick or disabled, causing them to be stereotyped (19). They also discover that mainstream media typically employ rhetorical methods to persuade the audience to agree with particular views and assertions, but do not mention or compare alternative viewpoints.

The lack of medical expertise among mass media reporters challenges the authenticity and objectivity of public health reporting content. Nelkin (21) notes that the majority of journalists and editors involved in mass media communication lack a medical background, despite the fact that public health is replete with medical terminology. This has resulted in an irreconcilable divide between the journalistic professionalism espoused by the media and the medical professionalism required by public health issues. Media coverage of health issues emphasizes narrative approaches but undervalues statistical data analysis (22). As authoritative sources for the content of a report, reporters typically rely on the judgment of doctors and medical professionals rather than publicly available records. The media do not instruct the public on how to evaluate the credibility of these referenced doctors and medical experts (23). In addition, the reporting bias of professional journalists and citizen journalists is also one of the controversial points in mass media reporting on health issues. According to Tong (24), in China, professional journalists in news organizations refer to specialized practitioners who have received professional training or studied journalism and communication and have successfully obtained a journalist certificate. The content of their reports represents the interests of news organizations. Grassroots journalists and citizen journalists emphasize citizen witnesses who have not received special news reporting training and witnessed the facts at the news scene. Their reports are more regarded as a spontaneous behavior of paying attention to social hot spots. Regarding news reports on doctor–patient conflicts, professional journalists often cannot arrive at the news scene immediately after the conflict occurs to record the conflict process; and citizen journalists’ reports may incorporate subjective emotional tendencies, and the objective attributes of the news are questionable (25).

## 3 Media reporting of the doctor–patient relationship in China

Numerous doctor–patient disagreements and their detrimental effects on the doctor–patient relationship have caught the attention of Chinese media. The media is replete with coverage of the doctor and patient dilemma, including medical conflicts and violent attacks on doctors (26). Statistics compiled by Pan et al. (2) reveal that from 2003 to 2013, the Chinese media documented 101 instances of doctor–patient confrontations encompassing all provinces. The School of Journalism and Communication at the Renmin University of China (RUC) compiled data on injuries and medical occurrences reported by mainland Chinese media from 2009 to 2018. It was discovered that the media has covered 295 injuries and medical incidents during the past decade (27). While the media is focusing on the doctor–patient conflict, the influence of the media on the formation of the doctor–patient relationship has become a hot topic in the academic community. According to Wang et al. (28), the discursive and rhetorical consequences of media coverage of doctor–patient disagreements are the primary cause of the strained doctor–patient relationship. Sun et al. (29) note that mainstream and internet media coverage of doctor–patient disagreements has a negative effect on patients’ and physicians’ perceptions of the doctor–patient relationship. In media stories, patients are described as vulnerable populations. With feelings of sympathy for the vulnerable population, the media provides support for patients and condemns hospitals and physicians.

A review of past studies reveals, however, that they frequently point out the subjective influence of the media on the creation of the doctor–patient relationship. They lack specific empirical proof. Even the 10-year statistical studies undertaken by Pan et al. (2) and RUC (27) provide only a brief description of the frequency of media coverage on doctor–patient confrontations or patient violence against doctors. They identify the reasons of doctor–patient conflicts and provide future recommendations for the development of a more harmonious doctor–patient relationship. They lack evidence, however, to corroborate their interpretation of the characteristics of media stories on doctor–patient confrontations. With the exception of RUC (27), the majority of past studies are rooted in medical and public health hygiene or management research. There are few investigations from the standpoint of media and communication studies. The majority of academics assert that the portrayal of doctor–patient dispute in the media deteriorates the doctor–patient relationship. However, few studies examine the function of mass media in the formation of the doctor–patient relationship. On the basis of these constraints and research gaps, this project examines modern Chinese media coverage on doctor–patient relationship difficulties from the perspectives of audience studies and stakeholders. It examines physicians’ and patients’ perceptions of citizen journalism on doctor–patient relationship concerns.

## 4 Methodology

According to Hansen and Machin (30), qualitative research methodologies are required to analyze the dynamics of the audience and whether they comprehend the framework of the media material, as well as the role the media plays in the daily lives of the audience. Brennen (31) believes that, among the numerous qualitative research methods, interviews (i.e., one-on-one or group interviews) are suitable for assessing the level of audience comprehension, use, and interaction with media content. Interviews were deemed appropriate as a qualitative research strategy for this study.

For interviewing doctors, the researcher chose semi-structured interviews (one–one interview as the research method). Due to the demands of their profession, the majority of doctors are extremely busy every day, making it impossible to schedule group interviews (such as focus groups). In these semi-structured interviews, the questions may be guided by an interview guide that allows for greater flexibility during the interview. This ensured that the doctor participants were able to communicate their understandings of citizen journalism in a flexible and in-depth manner while avoiding work-time constraints.

As a research approach for patient interviews, the researcher picked focus group. The focus group approach involves selecting a certain number of observational objects from the study population to form the sample, followed by conducting in-depth group interviews with sample members. Thus, based on the information from the samples, researcher was able to infer the population’s general features (32). In comparison to the medical participants, who all came from the same professional structure, the patient participants’ backgrounds were more complex. For example, they belonged to different social classes, were afflicted with different ailments, and had diverse work and home settings. Also, in regards to doctor–patient confrontations in China, some patients are more radical, exhibit activist behaviors, and present activist arguments than professionals (33). Therefore, adopting focus groups afforded them the chance to discuss and share their perspectives on the impact of citizen journalism on the doctor–patient relationship.

### 4.1 Semi-structured interviews with doctors

The criterion for selecting interviewees was based on the categorization of various hospital operational models in China. According to Yang (34), there are two sorts of operational models for Chinese hospitals: publicly supported hospitals and privately operated hospitals. A publicly funded hospital is one that is organized by the government and whose costs are included in the government’s budget. Therefore, a publicly funded hospital is state-owned. A privately operated hospital is a hospital that is not owned by the government and is managed by the private sector. In China, the majority of privately owned hospitals are for-profit, user-fee-funded medical institutions. Prior research demonstrates that both academia and the media view the marketization of healthcare reforms as a significant influence in transforming the identities of both physicians and patients, as well as a contributor to the formation of the doctor–patient relationship (35–37).

With the introduction of market-based healthcare reforms in the 1980s, China’s hospital system underwent tremendous transformation. It evolved into hospitals that are publicly funded and privately operated (34). Doctors are essential hospital and medical institution spokespeople. Therefore, it was fair to use the hospital’s operational model as the sample criterion for classifying the doctors who were interviewed. The researcher analyzed the interaction between marketization healthcare reforms, media reporting, and the doctor–patient relationship by assessing the similarities and differences between doctors from different types of hospitals regarding the impact of citizen journalism on the doctor–patient relationship based on interviews with doctors.

Compared with the United Kingdom and other Western developed countries which give full medical insurance coverage to citizens, and citizens can enjoy the free public medical services paid for by national tax revenues (38), Chinese health insurance is different. The cost of health insurance in China is covered by the state, business, and individuals. It includes three main types: one is labor insurance (lao bao) which applies to the state enterprise workers; the second is public healthcare insurance which is paid for by government and applies to the administration’s officers (shi ye dan wei gong zuo ren yuan); the last one is cooperative medical insurance (nong cun ju min he zuo yi liao bao xian) which applies to residents from rural areas (39). With the depth of medical reform, the state increased their reimbursement ratio into the personal health insurance, thus reducing the medical expenses of residents. However, Du (40) utilizing big data analysis reveals the relationship between Chinese economic reforms and health insurance coverage. He argues that with the structural update of the Chinese economy, a large number of state-owned enterprises and collective enterprises facing market competition have to lay off workers and reduce welfare benefits. This leads to the workers losing public health insurance. Despite the fact that they can be covered by the private insurance, they still need to pay more for treatment than before. Meanwhile, for citizens, there are some limitations in the reimbursement range of health insurance. The reimbursement ranges of health insurance in China only cover hospitalization costs, and most of the diagnostic tests, prescription drugs, and outpatient visits are not listed (41). This leads to a situation where the actual medical expenses of citizens remain high. China’s existing insurance system has been severely challenged by stringent reimbursement caps (42).

Regarding the operational stages involved in selecting doctor interviewers, they were first required to be affiliated with sampling-eligible facilities. This study was conducted in Weifang, Shandong Province, where the researcher has an established social network. Although the research objective of this project was to examine the interaction between citizen journalism and the doctor–patient relationship in contemporary China, the choice of Weifang as the research area may have created regional limitations. However, the geographical location was not a determining factor in classifying the doctors who were interviewed. Thus, this was the rationale behind picking several operational-model hospitals in Weifang for the purpose of recruiting qualified doctors for interviews. As sample hospitals for this analysis, the researcher picked three publicly supported hospitals and three privately owned hospitals. Weifang Renmin Hospital (H1), Weifang Centre Hospital (H2), and Weifang Cardiology Hospital (H3) were the three publicly supported hospitals; the three privately operated hospitals were Weifang Zunyi Hospital (H4), Weifang Guangming Hospital (H5), and Weifang Renkang Hospital (H6).

In each hospital, the researcher interviewed four doctors; so, there were 24 doctor participants in the one-to-one semi-structured interviews. Table 1 shows the details.

**Table 1:** The recruitment of doctor interviewees.

Hospital number	Hospital operational model (property)	Interviewee numbers and interviewee reference number	Total number interviewees
H1	Publicly funded	4 (101–104)	24
H2	Publicly funded	4 (201–204)	
H3	Publicly funded	4 (301–304)	
H4	Privately operated	4 (401–404)	
H5	Privately operated	4 (501–504)	
H6	Privately operated	4 (601–604)	

### 4.2 Focus groups with patient

Patient selection was based on the method Gong and Jackson (43) used for their research, using social status (defined by income) as the criteria to classify the patient into different groups. Gong and Jackson (43) utilized focus groups to investigate how new parents and grandparents understood infant formula advertisements and how this decoding was a component of their everyday risk management in China. Gong (44) utilized focus groups to examine parents’ reception of the discourse and their responses to perceived and actual health risks pertaining to childcare in China. Both Gong and Jackson (43) and Gong (44) highlight the public health challenges in China, which are situated in a comparable sociocultural framework to this project.

In this initiative, the participant’s income is based on the level of economic development in his or her neighborhood, rather than the individual’s own income. The focus groups were organized in the Shandong province city of Weifang. Choosing this variable to split the patient participants was primarily motivated by the need to connect insights gathered from the literature study with the project’s research aims. As above mentioned, contrary to the universal free medical insurance policy prevalent in Western nations (such as the United Kingdom), modern Chinese society has chosen paid medical service as its policy (39). For paid medical services, residents’ income cannot be neglected as a vital factor. It impacts how patients choose which hospital level to visit and the extent and quantity of compensation they can anticipate from medical insurance after treatment (33). Therefore, the researcher used patient income as the major criterion for categorizing patients who participated in focus groups.

For participant income level in Weifang City, the researcher divided the participants into different income level groups based on the 2017 *per capita* GDP ranking of each district in Weifang City (45). For example, Weifang’s *per capita* GDP in 2017 was 67,258 yuan. The researcher divided the participants’ income level based on the above average amount and each district’s *per capita* GDP in Weifang city: the patients with high incomes group refers to participants from the districts of Kuiwen, Hanting, Shouguang, and Zhucheng; the patients with middle incomes group refers to participants from the districts of Changyi, Gaoming, Qingzhou, and Weicheng; and the patients with low incomes group refers to participants from the districts of Changle, Fangzi, Anqiu, and Linqu.

In this project, the focus group participants were divided into nine groups, each type of income level of patients took up three groups. Every group had four patient participants. There were 36 patient participants in total. All the eligible patient participants had to be above the age of 18 and without life threaten physical illnesses, infectious diseases, or serious mental illnesses. The detailed sampling plan is shown in the following Table 2.

**Table 2:** The recruitment of patient participants.

Group number	Participants	Number of participant	Total participant numbers
F1	Patient with low incomes (F1A–F1D)	4	36
F2	Patient with low incomes (F2A–F2D)	4	
F3	Patient with low incomes (F3A–F3D)	4	
F4	Patient with middle incomes (F4A–F4D)	4	
F5	Patient with middle incomes (F5A–F5D)	4	
F6	Patient with middle incomes (F6A–F6D)	4	
F7	Patient with high incomes (F7A–F7D)	4	
F8	Patient with high incomes (F8A–F8D)	4	
F9	Patient with high incomes (F9A–F9D)	4	

During interviews and focus groups, the researcher employed the following interview and focus group format: Initially, the researcher posed a few questions to determine the participants’ overall impressions about the doctor–patient relationship, particularly citizen journalism on doctor–patient relationship concerns. The researcher then permitted the participants to read instances of citizen journalism authored by citizen journalists about doctor–patient conflicts. For instance, the researcher picked eight examples of citizen journalism as a sample to present to doctors and patients during interviews (the citizen journalism sample had been printed and given to each doctor and patient). The selection criteria for sample cases were based on Zhu and Yuan’s (72) classification of doctor–patient conflicts in China. Zhu and Yuan (72) divide the main doctor–patient conflicts in China into four conflict types based on what provokes them: *medical-expense/bribery*, *medical technique*, *medical outcome*, and *medical-service attitude*. Zhu and Yuan (72) describe the conflicts labeled medical-expense/bribery as disputes in which doctors and patients are at odds over medical-treatment fees. The conflicts labeled medical technique refer to conflicts over medical professionalism. In these conflicts, patients have suffered from negative, medical results either due to doctors’ malpractice or the patients’ belief that their doctors had committed malpractice. The conflicts categorized as medical outcome conflicts refer to disagreements about the quality of healthcare where the dissatisfaction of patients toward doctors is due to the patients’ beliefs that their medical treatments did not achieve their expected outcomes. The conflicts named medical-service attitudes conflicts center on questions of medical ethics. This points out how patients feel doctors had negative attitudes during medical treatments, thus triggering contradictions between doctors and patients. Each of the four type conflicts was represented by two of these eight pieces of citizen journalism. Following that, the interview and discussion focused on both their understandings of the eight citizen journalism issues and their actual hospital-related work or visit experiences. The interview and discussion elicited their individual perceptions of the stimulus material (i.e., the eight pieces of citizen journalism) and their understandings of the doctor–patient relationship that emerged after reading the stimulus material. All the interviews with doctors and focus groups with patients were voice-recorded. After each session was completed, the researcher transcribed the voice recording to text, and then did a thematic analysis on the transcripts directly based on the interview questions.

### 4.3 Ethical considerations

The ethics application for this project was approved by the Media, Communication and Sociology Research Ethics Committee, University of Leicester. During the ethics application, the researcher attached all key documents which included the hospital gatekeeper letters, information sheets, consent forms, and interview questions.

For the recruitment and interviews with doctor interviewees and patient participants, as they took place in the hospital environment, to guarantee the implementation of the interview activities, before the ethics application was submitted, the researcher sent the Chinese version of the hospital gatekeeper letter to the management team member of each hospital to explain in detail what the researcher would do in their hospital and how the researcher would consider the ethical issues during the interviews. Once the researcher received the approval from those hospitals, the researcher attached the signed and stamped hospital gatekeeper letters within the ethics application.

To guarantee this research did not affect the hospital’s daily operations, during the fieldwork in those hospital, the researcher hung a poster to introduce this project, which listed the requirements for doctor and patient participants, and attached researcher’s contact details. This let the doctors and patients who were interested in the project contact the researcher directly. For patient participants, the researcher specifically recognized the sensitivity of this group. Therefore, on the poster, the researcher listed that patients who were interested in participating in this research should be above the age of 18 and currently without life threaten physical illnesses, infectious diseases, or serious mental illnesses. When each of the patients expressed their interest in engaging in the focus group interview, the researcher asked their permission to contact their doctor. Then the researcher confirmed with the doctor in charge of their treatment whether the patient’s condition satisfied the above criteria. To protect the patient privacy, the researcher never ask those doctors the details of the health condition of the patient. Instead, the researcher showed the criteria of eligibility to those doctors, and let them determine whether each patient who was interested in the project could or could not engage the focus group interviews. Similarly, the researcher never asked for the health condition details for any of the patient during the focus group interviews, unless they presented their health conditions on their own. Even for this situation, the researcher still emphasized the anonymity of each patient and used a reference number instead for each patient in the data analysis chapter.

The researcher sent the Chinese version information sheets and consent forms to both doctors and patients before each of the interviews and focus groups started. The content of the information sheets and consent forms for the each of the above three groups was slightly different, in that they were only suitable for each participant type. The researcher specifically let them know they could feel free to quit the interview or focus group any time, even after they had signed the consent form.

The data collected from all interviews and focus groups was stored securely on a computer disk that was accessible only after the researcher entered his password. All the interview and focus group extracts listed in the later data analysis chapters are anonymized with a unique number. Any personally identifiable information provided by a respondent was treated confidentially in accordance with the 1998 Data Protection Act and the General Data Protection Regulation (GDPR) of the European Union. The respondent’s name and identifiable associations were handled anonymously in this research.

## 5 Results

### 5.1 Authority and witnessed as the core criteria to establish the trust in the reporting

The foundation of the doctor–patient relationship is confidence. Excellent communication between physicians and patients facilitates the development of mutual confidence (33, 46). This research focuses on determining which aspects of media reports contribute to the formation of doctors’ and patients’ perceptions of their credibility and the amount to which they trust citizen journalists to report on doctor–patient relationship concerns. The examination of interview and focus group data reveals that doctor and patient participants’ judgments of the credibility of citizen journalism are most influenced by authority and witnessing:

102: If it is reported by the doctor community, I believe it.

304: Quoting official authoritative sources is more trustworthy. Otherwise it would be nonsense if the reporter interviews a few people randomly. After all, if there is a doctor–patient conflict, we have to deal with it under the authoritative instructions issued from higher administrative departments, rather than the intervention of the media.

503: I believe those established citizen journalists.

Researcher: Any examples?

503: Like *Shao Shang Chao Ren A Bao* and *Yi Ge You Dian Li Xiang De Ji Zhe*. They have a sense of social justice and report many similar incidents.

Doctors attempt to rely on the opinions of medical colleagues, official releases, and influential citizen journalists to determine the credibility of these stories. They have more faith in the statements of their medical colleagues and specialists.

The majority of doctors interviewed held the same beliefs as doctor 102 (including 101, 104, 201, 302, 501, and 604). Although the majority of doctors interviewed indicated confidence in doctors as reporters or in their opinions as sources of quotations, the reporting methods of citizen journalists on doctor–patient relationship concerns do not fulfill doctors’ perceptions of authority and trust.

The comments of doctors 402 and 503 indicate that official information citations and the reporter’s authority are also significant markers of the credibility of citizen journalism. This shows the varying perceptions of the content’s and communicator’s authority among doctors interviewed. It ties these comprehensions directly to their own sociocultural contexts.

Doctor 304 is also the vice president of the hospital where he works, which is publicly funded. Personnel appointments for publicly financed hospital operations and administration are vulnerable to administrative intervention in China (34).

As an essential hospital executive, the vice president is responsible not just for the physicians and patients, but also for the administrative departments that oversee them (33). In this instance, these departments provide the vice president with directives for work arrangements that must be carried out. Therefore, it is not unexpected that the intervention of administrative power has endowed dual-identity physicians with a natural reliance on official, authoritative knowledge.

In contrast, junior doctor 503 from a privately operated hospital only relies on their recognition of reporters based on their understanding of authority and the trusting of their reports. The citizen journalists *Shao Shang Chao Ren A Bao* and *Yi Ge You Dian Li Xiang De Ji Zhe* were the two most active citizen journalists reporting on the cases of doctor–patient conflicts in China. Not only did they pay attention to the incident for a longer time span and post continuous reports, but the number of views and forward of their contents were also among the top of all reporters in the cases. Most of the reports they produced focus on the patients and patient families’ illegal behaviors during these conflicts. The two citizen journalists’ names are transliterated into Chinese pinyin. However, their original meaning in Chinese also reflects their self-identity. *Shao Shang Chao Ren A Bao* refers to the citizen journalist who is also an expert doctor in the burn department. In other words, his principal occupation is as a doctor, and he uses his spare time as a citizen journalist to report on doctor–patient relationship issues. In his name, the *Chao Ren* can translate as ‘superman’. It shows that this citizen journalist would like to show the public that he is a medical professional expert. This can also be seen as self-promotion for his reporting. He tries to get the audience to trust his authority and qualifications as a medical professional. *Yi Ge You Dian Li Xiang De Ji Zhe* can translated as ‘a journalist who has ideals’. Here the word ‘journalist’ can be seen as citizen journalist. This means this reporter recognizes his work is like what a professional journalist does. For the word ‘ideals’, it is difficult to see from his name what the ideals refer to here. However, by checking his daily reporting on his Weibo page, it can be found that he usually focuses on reporting public affairs. Especially, he prefers witnessing on the scene to let the audience know what has happened in a conflict.

These two citizen journalists’ understanding of and attentiveness to the medical community have satisfied doctor 503’s aspiration for the doctoring profession: to aid the injured without seeking compensation. Based on this, he labels these two citizen journalists as “accredited” and their stories as authoritative and persuasive.

The patient participants’ views of authority are more diverse than those of the doctors. They drew upon their own life experiences and social cognition to determine whether sources cited in citizen journalism may be considered credible authorities.

F7A: I trust the report from an authoritative person, or which quotes the opinion of an authoritative person.

F7C: Me too.

Researcher: Can we define what kind of person is an authoritative person you mentioned?

F7A: Those with high social status, or whose own social reputations or influence are high, like people who are working in government.

F7C: For example, 2 weeks ago, there was a doctor–patient conflict in Renji Hospital in Shanghai. The movie star Ge Hu made a speech on Weibo. Although he was not a witness, he forwarded the witness’s testimony. I would like to trust that, because Ge Hu is a movie star with a sense of justice. He never has scandals.

F2B: I think quoting the sources of mainstream media is definitely authoritative. The mainstream media represents the government stance. The government holds the power. It must not be nonsense. This is no joke.

F3C: I do not know who is an authoritative person, but I’m sure a report is not worth trusting if it quotes the doctor’s opinion. The doctor’s interest is in earning money from the patients. They will not sympathy with us even though we are the vulnerable group.

Respect for power and authority in Chinese culture and society is reflected in the patients’ understanding of authoritative figures. Patients themselves are susceptible to physical and emotional strains. In such a predicament, it is not strange that they place their trust in the government and the mainstream media, which serve the government. The aforementioned patients’ views of authority demonstrate that the patients’ confidence in authoritarian rule originates from their appreciation of their spokesperson status. According to F7A, government employees have high social standing, excellent social reputations, and considerable influence. According to F7C, this stronger social reputation and influence also applies to social celebrities, such as Ge Hu’s credibility on social media. Ge Hu is a well-known Chinese actor with 71.94 million Weibo followers and no scandals to his name. Ge corresponds to F7A’s conception of an authoritative figure.

However, patients exclude the group of physicians from the center of authority. Regarding the creation of the doctor–patient relationship, this indirectly reflects patients’ reservations in medical expertise and the efficacy of medical procedures employed by doctors. Such worries inhibit the growth of trust between physicians and patients.

On the second criterion for believing citizen journalism—seeing—doctors and patients expressed opinions that were comparable:

F1B: Citizen journalists must be at the scene of a doctor–patient conflict. I would not trust their report if they were not there.

F6C: Nowadays the reporting methods on the Internet are bizarre, and the Photoshop technology has created fake news pictures, often. Only if I see a citizen journalist witness the scene and use video reporting methods will I trust that reporting.

301: Medicine is a very rigorous scientific discipline. If citizen journalists do not witness the scene, how do they accurately report and disseminate the medical details?

504: I think the reporter is a conscientious reporter if they are reporting on the scene. They make me feel the final temperature on the cold-blooded doctor–patient conflict. Because at least, this reporter is willing to take the risk to record what happened in the conflict and spread it to the public.

Citizen journalism’s participatory reporting at the scene of an incident, according to Allan (25), is one of its essential communicative characteristics. Citizen witnessing has been the distinguishing characteristic between citizen journalism and traditional news media. When the government and mainstream media are unable to offer information on the progression of an occurrence during an emergency, citizen witnesses can successfully fill the information gap. Citizen journalists have intuitive and emotive emergency reporting experience, as opposed to the detached reporting style of the mainstream media. This experience is more meaningful to audiences (47, 48). According to Doctor 301, medicine is a logical life science. Conflicts between doctors and patients need that citizen journalists be present on the scene and collect firsthand witness materials for publication. In agreement with Robinson (47) and Vis (48), Doctor 504 argues that citizen journalists should convey the emotional impact of these situations in their stories.

Allan (25) argues further that citizen journalism that includes “citizen witnessing” promotes the propagation of a democratized news language that is basic, direct, and unquestionably subjective. If a citizen journalist sees an occurrence and transmits favorable eyewitness testimony on behalf of a doctor or patient, this testimony will be highly regarded by the audience of doctors or patients. This recognition is a result of their preference for this rudimentary and straightforward reporting language.

Shen (49) adds that in China, the reports of citizen journalists who act as eyewitnesses are more contagious and convincing than the reports of professional journalists who take an objective stance. This persuasive force is a result of this sort of citizen journalism replacing the impartial, model-style expressions of Chinese mainstream media news stories. These reports provide immediate access to first-hand proof of an incident for the audience. In reporting on the connection between doctors and patients, doctors and interviewees of patients are exposed to the influence and appeal of citizen journalist testimony. Doctors and patients’ trust in citizen journalism includes witnessing as a significant reference norm.

According to the doctor and patient participants, authority and witnessing are two essential criteria for the credibility of citizen journalism. It also investigates the parallels and contrasts between doctors’ and patients’ perceptions of the credibility of citizen journalism. In terms of attitudes and emotions, the interviews and focus group studies indicate that citizen journalist reports influenced the roles of doctor and patient participants. This will be covered in the next sections.

### 5.2 Concerns about the unfamiliar and uncertain knowledge

The results of the interviews and focus groups with doctors and patients indicate that their apprehensions regarding citizen journalism arise mostly from their apprehensions over unknown and ambiguous information offered in the reporting.

Patients’ concerns over the reports of citizen journalists originate mostly from their apprehensions regarding the potential health hazards posed by diseases. In addition, they are related to their unfamiliarity with medical diagnoses and treatment procedures. As a result of receiving the citizen journalist’s report, their sense of apprehension has increased. A cross-analysis of the content of the focus group interviews reveals that the anxiety of patients varies with their income level.

Patient with low-income:

F1C: In some reporting, it sounds like the doctor has not treated the patients well, and the patients’ families are not willing to accept the results. It scared me when I heard it. You know I am almost illiterate. When I came to the hospital, I did not understand where the east and the west was. I am worried that doctors lied to me and made me lose money. More seriously, I am worried if I loose my life.

F2A: Burning paper money and setting off firecracker…… These scenes appear in citizen journalism from time to time, and they become what people are interested in listening to. I feel that many conflicts are caused by patients who are not familiar with the hospitalization process. However, we do not have enough money and no high educational background; who will answer us regarding these unfamiliar issues?

Patient with middle-income:

F5D: Listening to the reports of doctor–patient conflicts is a headache!

Researcher: Why do you feel this way?

F5D: Do not get sick. When you enter the hospital, you are a layperson. You do not understand any medical knowledge.

F5A: To be honest, I am worried too. But I know worry is useless. I’ll try my best to avoid it if the conflict is not related to me.

Patient with high-income:

F7D: I came to the hospital this time because my heart was uncomfortable. I know it may be because I have been under pressure recently. But there is a more important reason: I have seen the symptoms of chest pain triggering heart attacks often mentioned in citizen journalism. I am especially worried that one day I will encounter it, but I will not get timely treatment. So, whenever I feel uncomfortable, I do an ECG in the hospital.

F9A: My leg has been hurting for 5 months. I have seen 8 doctors in the past 5 months, and I still have not recovered completely. You know, so many doctors-patient conflicts have been reported by the citizen journalist. Isn’t it because doctors and patients do not trust each other? I cannot believe any doctors. So, I can only see a few more doctors and compare the treatments they prescribe, then decide for myself.

The concern of low-income patients is related to their interpretation of the image of doctors and patients in citizen journalism.

Prior to reading the sample citizen journalism, the majority of patient participants believed that citizen journalists typically characterized patients as vulnerable groups and as victims in doctor–patient conflicts. This perspective is directly linked to their daily self-identity. The payment-based medical paradigm established in current Chinese culture exerts actual economic pressure on low-income patients. On the neoliberal market, consumers can freely purchase health as a commodity based on their financial condition (50). With the influence of neoliberalism, commercialization, privatization, and individualization of health management are becoming increasingly prevalent in China (41). Therefore, many patients must devote a substantial portion of their income in medical care. Nevertheless, medicine is an imperfect science, and not all ailments can be healed entirely (33). This type of uncertainty-based health risk has exacerbated the concerns of low-income patients regarding the uncertain return on their diagnosis and treatment investments. When they observe citizen journalists reporting on doctor–patient conflicts, they subconsciously blame the disagreements to the powerlessness of patients brought on by economic pressure. They will consider their own circumstances, which will heighten their anxiety regarding the financial load of medical bills.

Based on the responses to questions F1C and F2A, as well as a review of the demographic information of the patient participants, it was determined that the majority of low-income patients are older adults, retired individuals; laid-off and unemployed individuals; urban migrant workers; and remote, rural residents without fixed incomes. The degree of education of these individuals is low. Their money and level of education hinder their understanding of the rapidly evolving commercial medical system. They are at a loss when confronted with the complex medical treatment procedures and professional medical terminologies that occur in the therapies.

This misconception is also associated with the expansion of digital healthcare. With the growing popularity of digital diagnosis and treatment processes in China, prominent hospitals have implemented innovative methods including online appointment scheduling, online consultations, and electronic medical record release. These metrics have enhanced the effectiveness of diagnoses and therapies (33). Although the majority of digital natives support these measures, the older adults and less educated members of the aforementioned low-income groups are also excluded. The emergence of this digital divide causes these individuals to be uncertain and inexperienced with digital diagnostic and treatment procedures, which raises their anxiety.

In addition, the disparity between urban and rural medical insurance coverage makes it impossible for rural low-income groups and migratory workers to obtain complete health insurance in metropolitan hospitals (36). This unquestionably heightens their concern for medical welfare protection.

In contrast, the neoliberal medical model offers a greater variety of service options for the medical care of high-income individuals. High-income patients can access more convenient and advanced diagnostics and treatments by paying greater medical costs, thanks to their substantial wealth and life savings. However, they are not totally satisfied by this convenience. They are dissatisfied with the current medical system and doctor–patient relationship. Citizen journalism makes people concerned about the unpredictability and unfamiliarity of the information required to interact with doctors. This issue is a direct result of their over focus on health hazards.

The analysis of focus group interviews reveals that, in contrast to low-income patients, high-income patients are more concerned with whether or not their potential health concerns have been identified in a timely manner. In order to eliminate these presumed health dangers, people are willing to pay greater costs, consult with more skilled doctors, and undergo more thorough medical examinations. As previously stated in F7D and F9A, citizen journalists are concerned about the health hazards and adverse bodily reactions induced by infectious diseases in their daily reports. High-income viewers frequently compare these symptoms to their own health issues, and if there is any doubt, they promptly seek medical help. Through unrestricted payment of medical bills, patients with significant incomes strive to achieve zero risk to their own health. In addition, the worry of high-income groups regarding diseases indicated in citizen journalist reports is strongly correlated with their level of informational sophistication.

Although Seale (10) have shown the importance of citizen science and health communication on social media, which can make medical knowledge more accessible via social media. As argued by Yang (34), medicine is a highly specialized life science, If the public only obtains health care knowledge through media reports, it will not allow them to make accurate and independent judgments on the internal mechanisms and diagnostic criteria of diseases. High-income groups’ deeply of awareness regarding health hazards compels them to regularly seek out medical professionals. They undergo a cognitive process of questioning, critiquing, screening, and accepting health information from media channels including citizen journalism and professional doctors. As F9A noted, she saw the doctor multiple times, but ultimately relied on her own judgment.

Also concerned about the health concerns identified by citizen journalism are patients from middle-income households. In contrast to low- and high-income patient groups, however, middle-income patient groups avoid and reject novel and uncertain health risks, as evidenced by the preceding discussion. According to the focus group interviews, they believe that the doctor–patient conflicts mentioned in the news reports are far from their own, and they firmly believe that these things will not happen in their own medical experience. Even if there is a risk of poor communication between doctors and patients, they said they do not want to preset these difficult scenarios in advance and will analyze the specific problems when they occur. The rejection of unclear risks is a result of their desire to live in the present and avoid thinking about the future.

By examining the demographic information of middle-income patients, it was determined that the majority of middle-income patients were between the ages of 25 and 40 and were not a high-risk category for common, chronic diseases. Due to the ethical restrictions of the focus group, I was unable to determine which diseases these patients sought treatment for at the hospital; however, talks with middle-income patients revealed that the majority of them viewed themselves as temporary patients. They were optimistic about their recoveries from their respective ailments. This confidence mitigates some of their concern around unknown health hazards. In addition, although they have shown indifference to conflicts between doctors and patients in citizen journalism, conflicts that do not involve their own interests, they have stated that they pay particular attention to issues pertaining to their own diseases that are reported by citizen journalists. The connection between health hazards and self-care has refocused their attention on the pertinent reports.

The doctors’ concerns over the reporting of citizen journalists are closely tied to the patient’s behavior. As stated at the outset of this section, in recent years the mistrust between Chinese doctors and patients has led to an increase in the frequency of violent acts perpetrated by patients against doctors (2). The recurrent occurrence of situations in which doctors are injured casts a shadow over the doctors’ professional persona:

104: Citizen journalists often report on patients violently hurting doctors. These reports sound scary. The patient stabbed the doctor abruptly, causing immeasurable losses.

304: Whenever doctors in our department see reports of such incidents of patients violently hurting doctors, they forward them to our department WeChat group. Everyone teases each other sometimes: “we can wear bullet-proof vests at work.” But jokes are only jokes. To put it bluntly, we are very worried, for fear that one day we will encounter a patient and be harmed by him/her.

502: The hospital is also a public place, but there is no security check for admission. In citizen journalists’ reports, experts often call for hospitals to set up security check systems. I do not understand why it cannot be realized? The long wait is worrying for us. If the doctor’s safety cannot be protected, who will protect the public’s health?

604: As mentioned in the citizen journalism, doctors are always at risk, because the doctor’s communication time with each patient may be as short as only 10 min in the outpatient clinic. The doctor does not know the patient’s social background and is not sure about his/her motives for coming.

The aforementioned conversations with physicians demonstrate that doctors’ input on citizen journalism content focuses mostly on their own occupational safety issues. As commercial medical care becomes more prevalent, patients’ expectations of medical care services rise. As a result of the fact that commercial medical care offers various diagnoses and treatment pathways for patients of varying economic levels to choose from. This has resulted in a strong polarization of diagnosis and treatment patterns in contemporary Chinese culture. The majority of patients will seek treatment in well-known, urban, general hospitals, and primary care facilities will not be recognized. This creates a substantial disparity in the frequency of consultations between large, urban, general hospitals and primary medical-care institutions. This discrepancy makes it more difficult for doctors in large, metropolitan, general hospitals to address the demands of their patients (35, 51).

The three public hospitals that supplied interviewees for this study are big, regional, general hospitals, while the three private hospitals are specialty medical facilities. In other words, their achievements in specific medical specialties are exceptional. Every day, a huge number of new patients visit these six hospitals, requiring doctors to continuously reduce the time spent with each patient in order to satisfy the medical needs of more patients. Under this flawed time management system, the majority of physicians interviewed indicated that they lacked sufficient time to comprehend the social history, underlying ailments, mental health, and family status of each patient. They lack familiarity and ambiguity regarding the specific circumstances of the majority of new patients. Therefore, when they read about doctor–patient confrontations or instances of doctors being wounded by patients in citizen journalism, they naturally wonder if they would experience similar scenarios at work.

The topic of instituting security checks at hospitals, raised by doctor 502, was also raised by a number of doctors interviewed (such as 102, 301, 402, 603). They added that they have regularly observed in citizen journalism calls for the installation of hospital security inspection systems. However, in fact, these systems are not implemented in the hospitals where they work. The disparity between their conception of citizen journalism and the reality they confront heightens their anxiety around the unpredictability of their own occupational safety.

After reading instances of citizen journalism, the majority of patients reported feeling more concerned about health concerns. This is mostly owing to the fact that the sample of citizen journalism included numerous doctor–patient confrontations that had happened over the past few years. This was especially true for the ‘Gauze Gate’ (Chinese: 潍坊纱布门事件; pinyin:wéi fāng shā bù mén shì jiàn) tragedy that occurred in their hometown of Weifang.

The ‘Gauze Gate’ case refers to in 2016, the gauze left in a woman after giving birth in Weifang, Shandong Province. According to doctors, the gauze was left in the abdomen of the woman to staunch her bleeding because she was suffering from a hemorrhage. But, during her recovery, the women held it was due to the hospital’s medical malpractice. Once the event was reported by news platforms, it drew the attention of China Central Television News Channel (CCTV News) which produced follow-up reports. Meanwhile, a wide-ranging discussion had started about whether the doctors’ medical technique was reasonable or not (52).

This type of occurrence occurring around them affects their emotions more. Simultaneously, the samples entailed exposing the doctor–patient conflict process, and citizen journalists utilized a variety of sensational language that made them concerned for the future of the doctor–patient relationship. Patients of all income levels brought up an intriguing point: in the process of seeking medical treatment, patients would prefer to lessen their anxiety about health risks by consulting a familiar physician.

Finding a familiar doctor reflects the importance attached to the social network in Chinese cultural traditions. In China, individuals seeking help from familiar people through their social network is called *zhao shuren,* ‘seek familiar people’, (Chinese: 找熟人; pinyin: zhǎo shú rén) or *zhao guanxi*, ‘find a social network’ (Chinese: 找关系; pinyin: zhǎo guān xì) (53), pp.52–53. These two Chinese phrases were also mentioned many times in focus group interviews with patients. Hammond and Glenn (54) point out that Chinese people use *guanxi* to deals with bottlenecks or difficulties in their lives. *Guanxi* seekers and donors are equal in terms of social structure. Because of the mutual assistance and payback advocated in traditional Chinese culture. *Guanxi* donors wish to receive repayment from *guanxi* seeking parties in the future. In this process, the two stakeholder parties of *guanxi* have formed a community of interests and mutual trust. This has also led to the emergence of *guanxi* becoming more important than regulations in the social ecology (54). Similarly, patients hope to use their social networks to find familiar doctors in a particular hospital to treat them. This process demonstrates their trust in familiar people. Focus group results show that patients believed this kind of trust could effectively alleviate their concern about health risks. However, through cross-analysis, it was found that their understanding of how to look for *guanxi* varied due to their different income levels and social backgrounds.

Patients with low incomes may have difficulty locating their primary care physician based on their economic level, social standing, and degree of power ownership. Therefore, people instantly eliminate themselves from the search for a familiar physician. This disparity between their aspirations and reality heightens their apprehension toward the doctor–patient interaction. For them, finding a familiar doctor for assistance is likely a pipe dream.

In contrast to the coexistence of expectations and concerns exhibited by low-income groups, the majority of middle-income patient responses displayed a more tranquil understanding of this unique hospital culture (looking for familiar doctors). They analyzed their social identities and noted that their very simple interpersonal networks pose a significant barrier to locating a familiar physician. In addition, as stated by F5D, they believe that these renowned physicians cannot reach guanxi parity by assisting a patient. Therefore, searching for a known physician becomes a type of unnecessary self-comfort for middle-income patients.

In contrast, the majority of high-income individuals routinely seek out known physicians during their regular hospital visits. In comparison to patients with low and intermediate incomes, patients with high incomes have greater social connections and resources. In China’s implementation of a payment-based medical system, patients can frequently select a doctor who meets their needs by raising their money. It is not unexpected that individuals demonstrate the aforementioned attitude toward locating a known physician. At the same time, as F7B and F7C point out, the high level of concern regarding health risks causes them to believe that if they are unable to find a doctor who is familiar with them, their treatment may be delayed, and this will cause a new health crisis because other people have adopted this method in competition with them. To avoid health hazards, finding a known physician becomes a routine: a risk management technique.

However, the doctor interviewers dispute the expectations of the aforementioned patient subgroups to find familiar physicians. During semi-structured interviews with physicians, the interviewer discussed with physicians their perspectives on patients’ expectations on locating a familiar physician for medical care. The majority of physicians consider that the patient’s views and behaviors pose a threat to the doctor–patient relationship and are not conducive to the establishment of a fair medical environment. They argue that not all patients have access to a familiar physician. This behavior will unquestionably impair the happiness of various sorts of patients while visiting a doctor and will ultimately exacerbate the conflict between doctors and patients.

303: Not every patient can find a familiar doctor, and not every doctor deeply wants to accept the patient’s request to find a familiar doctor. However, sometimes our work is just a personal relationship, and it’s difficult to refuse the request.

402: With the first time, there will be a next time. Patients feel that finding a familiar doctor is not difficult, so some people break the rules even more and ask doctors to make additional diagnoses and examinations for them. These diagnoses may not be included in the hospital admission system, but are just promises made by personal relationships. If the diagnosis is incorrect, patients may use it as evidence to accuse the doctor, which undoubtedly further exacerbates the tension in the doctor–patient relationship.

According to the above physicians’ viewing, they reported having seen situations in which known patients requested further care. Their perspective on these requests is complex. On the one hand, they are cognizant of the unfairness of such actions toward other patients who are unable to locate a physician who is conversant with their condition. On the other side, they find it difficult to deny requests, given the majority of patients originate from their social networks. The presence of such complicated emotions heightens their anxiety about getting along with patients. In addition, after reading the example citizen journalism, the doctors’ concerns about their own professional safety shifted to their career decisions and future growth. In addition, these concerns reveal their powerlessness in the face of the numerous difficulties they confront in their employment.

## 6 Discussion

Doctor–patient trust is the cornerstone of the doctor–patient relationship. Patients’ trust of doctors reflects their belief that doctors are honest and kind. They will not harm patients but will do their best to treat patients’ diseases (55). Patients are in a vulnerable situation, and they believe that doctors will care about their interests. For their own benefit, patients need to trust doctors with their private information and physical bodies (56). Doctors’ trust in patients reflects their notion that patients will follow the doctors’ advice and cooperate with treatment (57). Doctor–patient trust is a complex subject. The mutual trust between doctors and patients is affected by communication factors such as the level of interpersonal communication and the prevalence of medical knowledge (55). However, past studies have also shown that differences in sociocultural backgrounds can also lead to differences in doctor–patient trust. The behavior and appearance of doctors, the education level of patients, and the degree of familiarity between patients and doctors all contribute to the construction of the doctor–patient relationship (58).

Discovering which elements of these reports contribute to the shaping of doctor and patient participations’ perceptions of their trustworthiness and to what extent they trust citizen journalists to report on doctor–patient relationship issues of the reporting is also a focus of this research. Jian (33) shows that citizen journalism is one of the most commonly used media form which doctor and patient participants receive news regarding doctor–patient relationship issues. Therefore, it is here that doctor–patient trust may be influenced by this reporting. This argument is noted by Sun et al. (59). Based on a large-scale questionnaire analysis of doctors and patients carried out in 136 Chinese hospitals, they found that the long-term, negative perspective of the Chinese media’s reporting and communication of news about doctor–patient relationship issues has aggravated the mutual distrust between doctors and patients. The analysis of the interview and focus group data in this study shows that authority and witnessing are the two major factors in doctor and patient participants’ perceptions of the trustworthiness of citizen journalism.

Authority and witnessing are the two important standards for building the trust of these doctor and patient participants in citizen journalism, yet, they have different interpretations of authority. Doctor interviewees believe that quoting the opinions of medical experts in reports is most authoritative. In addition, the doctor interviewees believe that official sources and source confirmed by influential citizen journalists are also authoritative. However, the patient participants believe that it is not authoritative to quote the opinions of doctors and medical experts. Denying the authority of doctors and medical experts is not conducive to the establishment of doctor–patient trust, yet, patient participants believe that the opinions of persons with high social-status and social reputations (such as government officials and celebrities) are authoritative. Doctors and patient participants have more consistent interpretations of the standard: witnessing. They believe that citizen journalists who witness an entire incident on the spot are persuasive. However, there is not enough evidence to show that citizen journalism that meets the above authority and witnessing standard will definitely gain the complete trust of doctor and patient participants.

China’s medical system has undergone a transition from free medical care to marketized healthcare (34). With the expansion of hospital marketisation, the price of medicines has risen (60), and the over-treatment and over-prescribing of drugs has become a typical phenomenon (61–63). Additionally, there is a large urban–rural divide in medical insurance coverage and an unequal distribution of disposable medical resources (36, 64). This series of reasons has caused patients’ dissatisfaction with doctors and medical institutions to expand.

These social changes in the medical and health field have caused damage to the construction of good doctor–patient relationships. The distrust between doctors and patients has increased. It has led to various incidents in which patients have taken violent actions to injure doctors (2). These incidents damage the confidence of doctors and patients in rebuilding the doctor–patient relationship.

It has also made them increasingly concerned about their identities in the doctor–patient relationship (33). At the same time, these doctor–patient incidents were full of contradictions and controversial narratives, and they became the focus of various media reports (59). In particular, they were full of exaggerated and satirical reporting language, which satisfied people’s curiosity about doctor–patient conflicts to a certain extent, but this has also made doctor and patient groups more concern about the medical treatment experience in contemporary China. Given this, the construction of the doctor–patient relationship is even worse (65).

citizen journalism, as a media platform, is frequently used by doctors and patients. It has continued to pay attention to reporting the doctor–patient conflicts (33). Results from the interviews and focus groups with doctor and patient participants show that their concerns about citizen journalism mainly stem from their concern about unfamiliar and uncertain knowledge presented in the reporting.

Doctors’ and patient participants’ express concerns about uncertain and unfamiliar knowledge during medical treatments. For patients, after viewing relevant citizen journalism, their doubts and uncertainties about the potential health risks during medical treatments have created concerns. Such concerns vary with their income levels. The concern of the doctor interviewees mainly come from their own occupational safety. The many cases of doctor–patient conflicts reported by citizen journalists have made them doubt the identities and motivations of unfamiliar patients as well as their workplace safety.

After reading the examples of citizen journalism, the patients’ concerns about unfamiliar and uncertain health risks increased. Patient participants from different income levels all mentioned that using their social relationships to find a familiar doctor for medical treatment can effectively ease their concerns. But this research also shows that their views and expectations of finding a familiar doctor vary with their income levels and social statuses.

This practice of finding familiar doctors was refuted by most doctor interviewees. They argued that this practice undermines the hospital environment and fairness and is not conducive to the construction of good doctor–patient relationships. But they also mentioned that they often experience this situation in their lives, and the reality has exacerbated their concern. After reading the samples, the concern of doctor interviewees also extended to concern about career choices and future development. They mentioned that endless incidents of violence against doctors in citizen journalism have caused the doctor profession to face unprecedented challenges.

## 7 Conclusion

Using citizen journalism reports on the doctor–patient interaction as an illustration, this study demonstrates that various core audiences (such as doctors and patients) understand the same content differently. Even for the same audience type (such as patient groups), characteristics such as financial level and age influence their comprehension of identical communication content. Specific to doctor–patient relationships, the revelation of the various audience understandings of citizen journalism can assist citizen journalists in developing methods to meet the needs of their audiences. It will increase the role of the media in mediating the strained doctor–patient interaction.

Authority and witnessing are two crucial criteria for gaining the trust of these doctors and patients who participate in citizen journalism, but they have diverse conceptions of authority. Doctors interviewed felt that it is most credible to cite the opinions of medical specialists in reports. They consider authoritative both official sources and sources corroborated by influential citizen journalists. However, patient participants consider that citing the opinions of doctors and medical professionals is not authoritative. Denying the authority of doctors and medical specialists is not conducive to establishing doctor–patient confidence; yet, patient participants believe that the opinions of individuals with high social standing and social reputations (such as government officials and celebrities) are authoritative. The norm for witnessing is interpreted more uniformly by physicians and patient participants. They feel that citizen journalists who are present for the entirety of an incident are compelling. However, there is insufficient data to conclude that citizen journalism that satisfies the aforementioned authority and witnessing requirements will acquire the full trust of doctor and patient participants.

During medical procedures, doctors and patients express concern about unknown and new information. As a result of viewing pertinent citizen journalism, patients have worries and uncertainty regarding the potential health hazards associated with medical treatments. Such issues vary based on a person’s income level. The primary worry of the interviewed doctors is their own occupational safety. The numerous instances of doctor–patient conflict reported by citizen journalists have prompted them to question the identities and motivations of unfamiliar patients, as well as their workplace security.

As a result of reading the examples of citizen journalism, the patients’ anxieties around unknown and uncertain health risks grew. Patients of varying financial levels agreed that utilizing their social networks to locate a known physician for medical care might effectively alleviate their anxiety. However, this study reveals that their perspectives and expectations on locating a familiar physician vary according to their wealth and social status.

The majority of doctors interviewed disputed this practice of finding acquainted doctors. They suggested that this approach damages the hospital environment and is not favorable to the development of excellent doctor–patient interactions. However, they also noted that they frequently encounter this issue in their lives, which has heightened their fear. As a result of reading the samples, the respondents’ concerns extended to career decisions and future development. They stated that several instances of violence against doctors in citizen journalism have posed unprecedented issues for the medical profession.

This project provides a perspective for public communication research to explore public health issues from the dimension of communication content and audience. Most previous studies have focused on the connection between communicators and communication content in public health issues. They emphasize that non-professional reporters with various identities have different focuses on public health discourse (33, 66, 67). However, previous research has ignored the relationship between communication content and audiences. Taking citizen journalism reports on the doctor–patient relationship as an example, this research shows that different core audiences (such as doctors and patients) have obvious differences in interpreting the same content. Even for the same type of audience (such as patient groups), various factors, such as income level and age, affect their understandings of the same communication content. The finding for the different audience preferences is exactly what academia would expect on the of basis of many decades of research on media audiences (68–71); specific to doctor–patient relationship issues, the discovery about the different audience understandings of citizen journalism will help citizen journalists finds suitable strategies to satisfy their audience’s demands. It will further stimulate the media’s role to mediate the tense doctor–patient relationship.

However, this project still has some limitations regarding its analytical design. For the semi-structured interviews with doctors and focus groups with patients, the fieldwork was conducted in the researchers’ hometown of Weifang city and mainly relied on researchers’ local social network. The rise of commercial medical services and the increase in the proportion of patients’ medical payments caused by marketisation medical reforms are common phenomena in all regions of China (36). Therefore, in the research design stage, geographic factors were not considered as the primary basis for distinguishing the interviewees recruited. According to Li (36) and Wagstaff (64), marketisation medical reforms have caused a gap in the distribution of medical resources between urban and rural areas. During the recruitment of focus group participants in the six hospitals in Weifang, the researcher paid attention to the proportion of urban and rural patients. At the same time, the researcher recruited the doctor interviewees and patient participants from specific hospitals. Their opinions regarding the doctor–patient relationship have been high connected with their experience in that current hospital. They showed this during the interviews and focus groups. On the other hand, doctor–patient conflicts are a common phenomenon taking place in a variety of Chinese cities (27). The interview and focus group results also show that doctors and patients are generally familiar with this topic. In this project, there is not sufficient evidence to show that research on other cities will have significantly different results regarding the issues under investigation. However, in future relevant research, if there is a plan to explore how doctors and patients use digital media tools (such as citizen journalism) as pathways to engage in doctor–patient interactions, the consideration of the geographical factors may become necessary, as people who live in different tier cities may have different media literacies for taking part in health communication. The popularity of digital medical systems in different cities may also affect their convenience to take part in digital health to some extent.

In China, the doctor–patient connection is a complex social issue. It encompasses not just the humanitarian issues of medicine and sociology, but also the state’s political action and economic reforms. Regarding doctor–patient relationship difficulties, this study could not concentrate on the media’s coverage of all subtopics. Only chosen physician-patient conflict concerns were investigated. According to earlier research (2) and the findings of this empirical study, this is the area where the media reported the most on doctor–patient relationship concerns. However, future research should focus more on how the media covers different subtopics under doctor–patient interaction difficulties. For example, the doctor–patient connection contains positive energy. Concern for humanistic care content regarding doctor–patient relationships demonstrates how citizen journalists subscribe to the mainstream value orientation when reporting. The investigation of these materials will aid scholars in conducting a more critical discourse analysis of media reporting on physician-patient relationship concerns.

## Data Availability

The raw data supporting the conclusions of this article will be made available by the authors without undue reservation.

## References

[1] Wang Z Li J. The reason and countering measures of medical disputes. Chinese Commun Doctors. (2012) 35:409–10.

[2] Pan Y Yang XH He JP Gu YH Zhan XL Gu HF. To be or not to be a doctor, that is the question: a review of serious incidents of violence against doctors in China from 2003–2013. J Public Health. (2015) 23:111–6. doi: 10.1007/s10389-015-0658-7

[3] Hesketh T Wu D Mao L Ma N. Violence against doctors in China. BMJ. (2012) 345:25–7. doi: 10.1136/bmj.e5730 22960376

[4] He AJ. The doctor–patient relationship, defensive medicine and overprescription in Chinese public hospitals: evidence from a cross-sectional survey in Shenzhen city. Soc Sci Med. (2014) 123:64–71. doi: 10.1016/j.socscimed.2014.10.055 25462606

[5] Zhou P Grady SC. Three modes of power operation: understanding doctor-patient conflicts in China's hospital therapeutic landscapes. Health Place. (2016) 42:137–47. doi: 10.1016/j.healthplace.2016.09.005, PMID: 27770670

[6] Costello DE. Health communication theory and research: an overview. Commun Yearbook. (1977) 1:557–68.

[7] Jackson LD. Information complexity and medical communication: the effects of technical language and amount of information in a medical message. Health Commun. (1992) 4:197–210. doi: 10.1207/s15327027hc0403_3

[8] Valente TW Saba WP. Mass media and interpersonal influence in a reproductive health communication campaign in Bolivia. Commun Res. (1998) 25:96–124. doi: 10.1177/009365098025001004

[9] Lupton D. Risk as moral danger: the social and political functions of risk discourse in public health. Int J Health Serv. (1993) 23:425–35. doi: 10.2190/16AY-E2GC-DFLD-51X2, PMID: 8375947

[10] Seale C. Media and health. London: Sage (2002).

[11] Lefebvre R. Social marketing and social change: strategies and tools for improving health, well-being and the environment. San Francisco: Jossey-Bass (2013).

[12] Wright KB O'Hair D Sparks L. Health communication in the 21st century. Malden, Mass: Blackwell Publishing (2008).

[13] Avery E. Contextual and audience moderators of channel selection and message reception of public health information in routine and crisis situations. J Public Relat Res. (2010) 22:378–403. doi: 10.1080/10627261003801404

[14] Leask J Hooker C King C. Media coverage of health issues and how to work more effectively with journalists: a qualitative study. BMC Public Health. (2010) 10:535. doi: 10.1186/1471-2458-10-535, PMID: 20822552 PMC2941688

[15] Schwitzer G Mudur G Henry D Wilson A Goozner M Simbra M. What are the roles and responsibilities of the media in disseminating health information? PLoS Med. (2005) 2:e215. doi: 10.1371/journal.pmed.0020215, PMID: 16033311 PMC1181881

[16] Martinson BE Hindman DB. Building a health promotion agenda in local newspapers. Health Educ Res. (2005) 20:51–60. doi: 10.1093/her/cyg104, PMID: 15253997

[17] Wallington SF Blake K Taylor-Clark K Viswanath K. Antecedents to agenda setting and framing in health news: an examination of priority, angle, source, and resource usage from a National Survey of U.S. health reporters and editors. J Health Commun. (2010) 15:76–94. doi: 10.1080/10810730903460559, PMID: 20390978 PMC3090661

[18] Len-Ríos ME Hinnant A Park S Cameron GT Frisby CM Lee Y. Health news agenda building: journalists’ perceptions of the role of public relations. Journal Mass Commun Quart. (2009) 86:315–31. doi: 10.1177/107769900908600204

[19] Lewis B Lewis J. Health communication: a media and cultural studies approach. Basingstoke: Palgrave Macmillan (2015).

[20] Watney S. Policing desire: pornography, AIDS and the media. Minneapolis: University of Minnesota Press (1997).

[21] Nelkin D. An uneasy relationship: the tensions between medicine and the media. Lancet. (1996) 347:1600–3. doi: 10.1016/S0140-6736(96)91081-8 8667872

[22] Larsson A Oxman AD Carling C Herrin J. Medical messages in the media – barriers and solutions to improving medical journalism. Health Expect. (2003) 6:323–31. doi: 10.1046/j.1369-7625.2003.00228.x, PMID: 15040794 PMC5060204

[23] Kline KN. A decade of research on health content in the media: the focus on health challenges and sociocultural context and attendant informational and ideological problems. J Health Commun. (2006) 11:43–59. doi: 10.1080/10810730500461067, PMID: 16546918

[24] Tong J. The formation of an agonistic public sphere: emotions, the internet and news media in China. China Inform. (2015) 29:333–51. doi: 10.1177/0920203X15602863

[25] Allan S. Citizen witnessing: revisioning journalism in times of crisis. London: Polity Press (2013).

[26] Tang L. The patient's anxiety before seeing a doctor and her/his hospital choice behavior in China. BMC Public Health. (2012) 12:1121. doi: 10.1186/1471-2458-12-1121, PMID: 23270526 PMC3536590

[27] RUC. Yihuan chongtu: shinian fuzhen [The Chinese doctor-patient conflicts in the past ten years]. (2019). Available at: https://www.sohu.com/a/299260082_649502 (Accessed March 2021) (In Chinese).

[28] Wang Y Wang Y Fang M. How to decrease violence against doctors in China? Int J Cardiol. (2016) 211:66. doi: 10.1016/j.ijcard.2016.02.154 26977580

[29] Sun J Wang J Liu S Liu Q Wang Z Chen Y. The impact of adverse media reporting on doctor–patient relationships in China: an analysis with propensity-score matching. Lancet. (2017) 390:S100. doi: 10.1016/S0140-6736(17)33238-5 PMC6104794 30121612

[30] Hansen A Machin D. Media and communication research methods. Second ed. London: Red Globe Press (2019).

[31] Brennen BS. Qualitative research methods for media studies. New York: Routledge (2012).

[32] Thomas G. How to do your research project. California: Sage (2009).

[33] Jian H. Yihuan weiji yu meiti guanxi yanjiu [Research on the relationship between doctor-patient crisis and media]. Nanjing: Southeast University Press (2014) (In Chinese).

[34] Yang J. Serve the people: understanding ideology and professional ethics of medicine in China. Health Care Anal. (2010) 18:294–309. doi: 10.1007/s10728-009-0127-y, PMID: 19787458

[35] Bhattacharyya O Delu Y Wong ST Bowen C. Evolution of primary care in China 1997–2009. Health Policy. (2011) 100:174–80. doi: 10.1016/j.healthpol.2010.11.005, PMID: 21145123

[36] Li L. The challenges of healthcare reforms in China. Public Health. (2011) 125:6–8. doi: 10.1016/j.puhe.2010.10.010, PMID: 21168176 PMC7118747

[37] Yip W Mahal A. The health care systems of China and India: performance and future challenges. Health Aff. (2008) 27:921–32. doi: 10.1377/hlthaff.27.4.921, PMID: 18607024

[38] Ross F. Questioning path dependence theory: the case of the British NHS. Policy Polit. (2007) 35:591–610. doi: 10.1332/030557307782453047

[39] Huang X. Expansion of Chinese social health insurance: who gets what, when and how? J Contemp China. (2014) 23:923–51. doi: 10.1080/10670564.2014.882617

[40] Du J. Economic reforms and health insurance in China. Soc Sci Med. (2009) 69:387–95. doi: 10.1016/j.socscimed.2009.05.014, PMID: 19520475 PMC7116972

[41] Gong Q. Children's healthcare and parental media engagement in urban China: a culture of anxiety? London: Palgrave Macmillan (2016).

[42] Le Deu F Parekh R Zhang F Zhou G. Healthcare in China: entering uncharted waters. Shanghai: McKinsey & Company (2012).

[43] Gong Q Jackson P. Mediating science and nature: representing and consuming infant formula advertising in China. Eur J Cult Stud. (2013) 16:285–309. doi: 10.1177/1367549413476013

[44] Gong Q. Communicating risk and protection: advertising discourse of young children’s healthcare products and parental reception in China. Eur J Cult Stud. (2016) 21:1–19. doi: 10.1177/1367549416656859

[45] Sohu (2018). 2017 nian shandong weifang shi renjun GDP paiming [Per capita GDP ranking of each district in Weifang city, 2017]. Available at: http://m.sohu.com/a/237090698_787336 (Accessed September 8, 2024) (In Chinese).

[46] Koirala N. Trust and communication in a doctor patient relationship. Birat J Health Sci. (2019) 4:770.

[47] Robinson S. If you had been with us': mainstream press and citizen journalists jockey for authority over the collective memory of hurricane Katrina. New Media Soc. (2009) 11:795–814. doi: 10.1177/1461444809105353

[48] Vis F. Wikinews reporting of hurricane Katrina In: Allan S Thorsen E, editors. Citizen journalism: global perspectives. New York: Peter Lang (2009). 65–74.

[49] Shen J. Zimeiti shidai de gongmin xinwen *[Citizen journalism: in the era of we media]*. Beijing: China Radio & Television Publishing House (2013) (In Chinese).

[50] Gaffney A. The neoliberal turn in American health care. Int J Health Serv. (2015) 45:33–52. doi: 10.2190/HS.45.1.d 26460446

[51] Li H Liu K Gu J Zhang Y Qiao Y Sun X. The development and impact of primary health care in China from 1949 to 2015: a focused review. Int J Health Plann Manag. (2017) 32:339–50. doi: 10.1002/hpm.2435 28670754

[52] Sohu (2016). Shabu men shijian, yangshi jizhe daoqian le [The journalist of CCTV apologised for the ‘gauze-gate’ event]. Available at: http://m.sohu.com/n/472643131/ (Accessed September 12, 2024) (In Chinese).

[53] Luo Y. Guanxi and performance of foreign-invested enterprises in China: an empirical inquiry. Manag Int Rev. (1997):51–70.

[54] Hammond SC Glenn LM. The ancient practice of Chinese social networking: Guanxi and social network theory. Emergence (Mahwah, NJ). (2004) 6:24.

[55] Pearson SD Raeke LH. Patients’ trust in physicians: many theories, few measures, and little data. J Gen Intern Med. (2000) 15:509–13. doi: 10.1046/j.1525-1497.2000.11002.x 10940139 PMC1495476

[56] Goold SD Lipkin M Jr. The doctor–patient relationship: challenges, opportunities, and strategies. J Gen Intern Med. (1999) 14:S26–33. doi: 10.1046/j.1525-1497.1999.00267.x, PMID: 9933492 PMC1496871

[57] Goold SD. Trust, distrust and trustworthiness: lessons from the field. J Gen Intern Med. (2002) 17:79–81. doi: 10.1046/j.1525-1497.2002.11132.x, PMID: 11903779 PMC1495000

[58] Gopichandran V Chetlapalli SK. Trust in the physician-patient relationship in developing healthcare settings: a quantitative exploration. Indian J Med Ethics. (2015) 12:141–8. doi: 10.20529/IJME.2015.043, PMID: 26228046

[59] Sun J Liu S Liu Q Wang Z Wang J Hu CJ. Impact of adverse media reporting on public perceptions of the doctor–patient relationship in China: an analysis with propensity score matching method. BMJ Open. (2018) 8:e022455. doi: 10.1136/bmjopen-2018-022455, PMID: 30121612 PMC6104794

[60] Liu X Hsiao WCL. The cost escalation of social health insurance plans in China: its implication for public policy. Soc Sci Med (1982). (1995) 41:1095–101. 10.1016/0277-9536(94)00423-q 8578332

[61] Chen X. Defensive medicine or economically motivated corruption? A Confucian reflection on physician care in China today. J Med Philos. (2007) 32:635–48. doi: 10.1080/03605310701681021 18027252

[62] Dong L Yan H Wang D. Antibiotic prescribing patterns in village health clinics across 10 provinces of Western China. J Antimicrob Chemother. (2008) 62:410–5. doi: 10.1093/jac/dkn153, PMID: 18400805

[63] He AJ Qian J. Hospitals' responses to administrative cost-containment policy in urban China: the case of Fujian Province. China Q. (2013) 216:946. doi: 10.1017/S0305741013001112

[64] Wagstaff A. Reforming China's rural health system. Washington, D.C: World Bank Publications (2009).

[65] Zhou M Zhao L Campy KS Wang S. Changing of China׳s health policy and doctor–patient relationship: 1949–2016. Health Policy Technol. (2017) 6:358–67. doi: 10.1016/j.hlpt.2017.05.002

[66] Davis S. Citizen health journalism: negotiating between political engagement and professional identity in a media training program for healthcare workers. Journal Pract. (2017) 11:319–35. doi: 10.1080/17512786.2016.1230022

[67] Yang J Hong Y Ma S. Impact of the new health care reform on hospital expenditure in China: a case study from a pilot city. China Econ Rev. (2016) 39:1–14. doi: 10.1016/j.chieco.2016.03.005

[68] Faber RJ O'Guinn TC Meyer TP. Diversity in the ethnic media audience: a study of Spanish language broadcast preference in the U.S. Int J Intercult Relat. (1986) 10:347–59. doi: 10.1016/0147-1767(86)90017-9

[69] Knobloch-Westerwick S. Choice and preference in media use. Milton Park: Taylor and Francis (2014).

[70] Livingstone S Lunt P. The mass media, democracy and the public sphere In: Livingstone S Lunt P, editors. Talk on television: audience participation and public debate. London: Routledge (1994). 9–35.

[71] McQuail D. Audience analysis. London: Sage publications (1997).

[72] Zhu L Yuan Y. ‘Xian jieduan woguo yihuan maodun de leixing, tezheng yu duice [The Types, Characteristics and Solutions of Doctor-patient Conflicts in Contemporary China]. Social Science Research. (2014):104–111 (In Chinese).

